# A mixed methods study of hope, transitions, and quality of life in family caregivers of persons with Alzheimer's disease

**DOI:** 10.1186/1471-2318-11-88

**Published:** 2011-12-22

**Authors:** Wendy D Duggleby, Jennifer Swindle, Shelley Peacock, Sunita Ghosh

**Affiliations:** 1 Faculty of Nursing, University of Alberta, Edmonton, Canada; 2 College of Nursing, University of Saskatchewan, Saskatoon, Canada; 3 Alberta Health Services-Cancer Care, University of Alberta, Edmonton, Canada

## Abstract

**Background:**

Several research studies have reported the poor quality of life of family caregivers of persons with Alzheimer's disease (AD). However, factors that influence their quality of life have not been clearly defined. The purpose of this study was to examine factors associated with the quality of life of these caregivers such as demographic variables, their transition experience, and hope. A secondary aim was to explore the transition experience of family caregivers of persons with AD.

**Methods:**

A cross-sectional triangulation data transformation model mixed method design (Quant +Qual) was utilized to address the purpose of the study. Eighty family caregivers of persons with AD completed a survey with quantitative measures [demographic variables, Herth Hope Index (HHI-hope), World Health Organization Quality of Life -BREF (WHOQOL-BREF)] and a qualitative survey about their transitions experience. The qualitative data (transition open ended- survey) was converted to quantitative data using content analysis. Variables significant at the p < 0.10 level in the univariate analysis were entered in the multivariate generalized linear model used to determine significant factors associated with quality of life.

**Results:**

Subjects with higher hope scores (p < 0.0001) (Factor 1: temporality and future-cognitive-temporary dimension of hope) and who dealt with their transitions by actively seeking out knowledge and assistance (p = 0.02) had higher overall quality of life scores. HHI scores were associated with overall quality of life and for each of the four quality of life domains (physical psychosocial, relationships, and environment).

**Conclusions:**

Hope played a significant role in the subjects' perceptions of overall quality of life as well as the 4 quality of life domains. This underscores the need to develop ways to foster hope in family caregivers. Moreover, the active engagement of families in seeking information and help, as a way to deal with their transitions, suggests encouraging this engagement is important. The findings of this study also suggest many directions for future research, such as increasing our understanding of the processes of transitions for this population.

## Background

The World Health Organization has predicted that 34 million people will be diagnosed with dementia by the year 2025 and the majority of their care will be provided by family members [1]. Studies of caregivers caring for persons with chronic illnesses suggest that the experience of care giving can affect their physical and mental health [2-5]. Physical health outcomes include a decrease in immune system response, increased risk of cardiovascular disease and high blood pressure [2] and interruptions in sleep [3]. Caregivers have higher rates of affective and anxiety disorders [6] and depressive symptoms [2] than non-caregivers. Often they have a poor quality of life, which is a predictor of nursing home placement for their relative with dementia, as well as their own utilization of health care services as a consequence of their poor mental and physical health [7,8].

Very little research has focused on quality of life of family caregivers of persons with dementia. A common element in the definitions of quality of life used these studies was the individual's perception of general wellbeing which included physical, psychological, and financial well-being [9,10]. In a phenomenological study of 32 family caregivers of persons with Alzheimer's disease (AD) exploring the meaning of quality of life, factors influencing the participants' quality of life were described as: 1) the health of the person they were caring for, 2) independence of the person they were caring for, 3) help with caregiving, and 4) worries about the future [10]. Other quality of life studies with family caregivers of persons with dementia found demographic variables such as relationship to family member [11], length of the time care giving [12], and hours of informal care [13] were significantly associated with quality of life.

Multiple concurrent transitions also influence the quality of life of caregivers of persons with dementia [9]. Transitions are processes triggered by change [14,15] during which a new situation or circumstance is incorporated into their lives [16]. Transition theory [14] suggests that transitions are processes of change which include how a caregiver deals with their transitions and what influences them. Studies of transitions of caregivers of persons with AD suggest that there is a relationship between transitions and quality of life [9,17].

The relationship between transitions and the quality of life of family caregivers of persons with dementia was the focus of Bond and colleagues' study [9]. Quality of life was compared among three groups of family caregivers of persons with dementia: those continuing to give care in their homes, those with a person in a long term care facility and those who were bereaved. These three groups were chosen to represent the major transitions in the population of study. The groups did not have significantly different overall quality of life scores, but positive changes were evident in psychological well-being and activity in the groups no longer caring for a person at home. This suggests a possible relationship exists between transitions and aspects of quality of life. A limitation of this study is that other significant transitions experienced by family caregivers of persons with dementia and their relationship with quality of life were not examined. One study describing transitions experienced by 20 family caregivers of persons with dementia suggested that multiple transitions occur [17]. These were described as the family member taking on new tasks (instrumental tasks, decision-making, interactive nature, emotional cheerleader, negotiating care), changes in relationships, increases in negative emotions, changes in support from others and thoughts of the future; hopes and hesitations [17]. Hope was also described by the study participants as influencing their quality of life.

Hope has been found to help family caregivers deal with the challenges of caregiving [18]. In a qualitative study of 17 family caregivers of persons with dementia, the participants described hope using the metaphor of a knot, as it kept them from sliding into despair [18]. In another study of 80 caregivers of persons with dementia hope was found to mediate the relationship with stress and quality of life [19]. Thus hope is a psychosocial resource that helps family caregivers deal with transitions and the challenges of the caregiving experience [18] and has been found to be a factor in their quality of life [19].

These studies suggest that caregiver demographic variables, transitions and hope may influence the quality of life of family caregivers of persons with dementia. A better understanding of the transitions experienced by caregivers of persons with dementia and their relationship with hope and quality of life is essential for the development of effective ways to support them during their care giving experience.

### Purpose

The purpose of this mixed method concurrent descriptive study was to examine the relationship among demographic variables, hope, quality of life and transitions of family caregivers of persons with AD. A secondary aim was to explore the transition experience which describes the types of transitions experienced by family caregivers of persons with AD, what influences them and how they deal with the transitions.

## Methods

A cross-sectional triangulation data transformation model mixed method design (QUANT +Qual) was utilized to address the purpose of the study. In this design, both types of data are given equal emphasis and collected simultaneously. As well, one type of data is transformed into other type with the intent to interrelate different data types [20]. In this study, both quantitative and qualitative data were collected at the same time and the qualitative data (transition data) was converted to quantitative data using Krippendorff's [21] content analysis. Subjects provided consent by returning their surveys, and ethical approval was obtained from the University of Alberta Research Ethics Board.

### Sample

Using convenience sampling, subjects who were 18 years of age and older and caregivers of persons with AD were recruited. Caregivers were defined as a person who is providing assistance (e.g. emotional, financial, and physical) to a person with AD. Subjects were recruited through a provincial Alzheimer Society and Alberta Caregivers Association mail-out and some were recruited through support group sessions. The study required a sample size of 77 based on Cohen's [22] formula for determining sample size for regression, with a moderate effect size (f = .15), power of .080 and an alpha of 0.05 and three independent variables (demographic variables, hope and transitions). Quality of life, which is a measured on a continuous scale, was the dependent variable.

### Data collection

Five hundred surveys were mailed and distributed (November 2010-February 2011). The surveys included a letter of invitation to participate in the study, a demographic form, Herth Hope Index (measure of Hope), the World Health Organization Quality of Life - BREF (quality of life) and open ended survey questions on transitions. A prepaid postage envelope was also included and study subjects were asked to return the completed survey in the mail. Return of the surveys implied informed consent.

### Measures

#### Demographic form

Demographic data were obtained from the family caregiver including age, gender, marital status, ethnicity, education, occupation, income, and religious affiliation. Additional information was collected including relationship to person with AD (caree), length of time caring for caree. Information about the caree was also collected such as the age, gender and other medical diagnosis.

#### Herth Hope Index (HHI)

The HHI [23] was used to evaluate hope. The HHI is a 12 item 4-point likert scale that addresses three factors of hope: temporality and future, positive readiness and expectancy, and interconnectedness [23]. The former reflects questions such as believing each day has potential and having goals. Positive readiness and expectancy reflects questions such as seeing possibilities and having a sense of direction. Interconnectedness reflects faith, deep inner strength, giving and receiving care and love. This survey takes approximately 5 minutes to complete. Summative scores range from 12 to 48 with the higher total scores indicating greater hope. The HHI has been found to be reliable (test-retest r = 0.091) and valid (convergent validity, r = -0.84, criterion, r = 0.092; divergent, r = -0.73 [23]. Cronbach's alpha for the HHI in the present study was 0.75.

#### Brief version of World Health Organization Quality of Life (WHOQOLBREF)

The WHOQOL-BREF [24] is a quality of life assessment measure based on the World Health Organization definition of quality of life reflecting 4 domains of quality of life: physical health (Domain 1), psychological health (Domain 2), social relations (Domain 3), and environment (Domain 4). The WHOQOL-BREF is comprised of 26 items measuring the domains and is a shorter version of the original WHOQOL. The scores are not reported as a total, but per domain. The higher the score, the higher the subjects' reported quality of life in each domain. Question 1 of the WHOQOL-BREF is a question of overall quality of life using a likert scale with 5 being the highest quality of life. Question 2 is a measure of satisfaction of quality of life with 5 being most satisfied. This survey takes approximately 10 minutes to complete. Reliability has been reported to be 0.82 [24]. Cronbach's alpha for this scale was r = 0.76.

#### Open ended survey transitions questions

No reported reliable and valid measures of transitions are known to exist that are congruent with the definition of transitions as processes of integrating significant changes into a person's life. To capture transitions we thus employed five open-ended questions, used in a previous study on transitions [25]. The questions were: 1) please tell us about the biggest changes you have experienced in caring for your family member 2) how did you deal with these changes? 3) what do you think had an influence on the changes? 4) was there anything you think could have helped? and 5) anything else you would like to tell us?

### Data analysis

Demographic, HHI and WHOQOL-BREF data were entered into SPSS version 18 (PASW- IBM Company) and cleaned and checked. Open-ended survey responses were transcribed and entered into a NVIVO software program. As this was a transformative mixed method study, the qualitative data was transformed into quantitative data so that it was integrated at the analysis stage. The transformation of the qualitative data began using content analysis to identify themes for each question. This was accomplished by reading the answers from all participants to each survey question as a whole. Then data for each question were categorized into overall themes associated with the question. Themes for each question were then assigned a number and considered a variable. Numbers were entered into SPSS for each participant reflecting the most predominant theme of their answers to each individual question.

Spearman's rho correlation coefficients were calculated to determine significant relationships (p ≤ 0.05) for quality of life scores and ratio and interval data (i.e. hope, age, length of time care giving etc.). For categorical data a student's t- test was utilized (e.g. transitions, rural versus urban demographics and medical diagnosis of persons with AD etc.) to determine any significant relationships with quality of life scores. At the univariate level, generalized linear model (GLM) method was used to examine the relationships between the outcome quality of life with various covariates discussed above. Variables significant at p < 0.10 level in the univariate analysis were entered in the multivariate model. Variables that were significant in the multivariate generalized linear model at p < 0.05 level were considered factors that were associated with the outcome variable of quality of life.

## Results

### Sample

Ninety-three surveys were returned (response rate of 18.4%) with 80 completed by caregivers of persons with AD and the remaining 13 completed by caregivers of persons with other diseases and consequently did not meet the eligibility criteria for this study. The caregivers were typically older adults [Mean age 67.2 (SD 10.67)], educated [mean years of education 14.62 (SD 3.2)], female (83.8%), living in urban areas (71.3%) and the spouses of the person with dementia (65%). The majority were caring for persons with AD in their homes (75/80). On average they had been caregivers for 4.85 (SD 3.57) years (See Table 1 for demographic characteristics). The persons they were caring for were typically older [mean age 77.6 years (SD, 8.9)] and the majority were males [n = 48(60%); Female n = 32(40%)].

**Table 1:** Demographic characteristics of subjects N = 80

Demographic characteristic	n (%)
Gender	
Female	67 (83.3)
Male	13 (16.2)
Rural or urban	
Rural	21 (26.3)
Urban	57 (71.3)
Missing	2 (2.5)
Relationship to caree	
Husbands	12 (15)
Wives	40 (50)
Children	24 (30)
Other	4 (5)
Annual income	
Under $ 40,000	16 (25.4)
Over $40,000	51 (74.6)
Missing	13 (16.2)
Ethnicity	
Caucasian	78 (97.5)
Other	2 (2.5)
Marital status	
Married	71 (88.8)
Divorced	3 (3.8)
Widowed	3 (3.8)
Single	2 (2.5)
Common-law	1 (1.3)
Religious preference	
Catholic	10 (13)
Non-Catholic	44 (55)
Other	10 (13)
None	13 (16.3)
Missing	3 (3.8)
Anyone else helping with care giving?	
No	35 (43.8)
Yes	42 (52.5)
Missing	3 (3.8)

### Transitions

The words of the subjects were utilized as much as possible in the themes for the open ended questions in the written survey (see Table 2 for themes and data examples). Five themes emerged in the subjects' responses to the question regarding the biggest changes they experienced in caring for their family member: a) being consumed by responsibility, b) negotiating care for their caree, c) feeling isolated, d) decreases in their own physical and mental health and e) personal growth towards becoming a better person. Themes from the question regarding how did they dealt with the changes were: 1) taking one day at a time, 2) actively seeking knowledge and assistance, 3) connecting with other family members and friends, 4) learning to rely on one-self and 5) very negative emotions which resulted in anger, and crying.

**Table 2:** Transitions: themes and data examples

Question	Response themes and data examples
**Please tell us about the biggest changes you have experienced in caring for your family member?**	Consumed by responsibility: *"The most overwhelming part of care giving is the 24/7"; "Full time job..."* *" I am consumed and overwhelmed"* Personal growth:*"I am a very different person..."* *"I had to develop new skills and patience..."* Negotiating help: "*I had to spend considerable time with the many caregivers in helping them get to know her needs, likes and dislikes..."* Roles and relationships:*"Change in role. I am now my parent's main support rather than them being mine..."* Decrease in own health:*"Lack of sleep... Depression, anxiety and despair..."* Feeling isolated from or abandoned by friends and family:*"... I just feel so alone..." "My friends and family just have left me to do it all"*
**How did you deal with these changes?**	One day at a time: *"Taking things one day or one issue at a time helps me to avoid feeling overwhelmed..."* *"I try to go with the flow..."* Negative emotions: *"Not very well at first... I was upset... I cried... I lost my temper..."* Actively seeking knowledge and assistance:*"I tried to learn everything I can about the disease..." "First, I asked for help from anyone I could think of..."* Connecting and looking to other family members and friends:*"Sharing with immediate family..." "LOTS of communication with family and friends..."* Self- care:*"I try to take care of my own health..."*
**What do you think had an influence on the changes?**	Lack of any one else*: "I have no choice" "... no one else came forward*". Acknowledging importance of self care: "*I recognized that I had the Right to take care of myself. This was not a selfish act - it enabled me to be a better caregiver"* Love for another:*"Deep caring between my wife and I*." The disease process: "*My husband's diagnosis and the progression of his disease." "The illness influenced change"* Access to information:*"Learning about the disease helped me to cope with his symptoms and to keep him safe." "Our involvement with the Alzheimer's society has been an invaluable source of information*."
**Was there anything you think could have helped?**	Support of family and friends: "*The continued support of my family and friends"* Access to appropriate services and knowledge: "*An individual must know all the resources available and how to access them*. " Empathy, understanding and knowledge by health care professionals: "*The staff on the whole could have tried to be more understanding. I would write a book about the lack of care and caring."*
**Question 5: **Anything Else?	Frustrations abound: "*I feel frustrated and alone*". Feeling grateful: "*I am so lucky, I am Blessed with very nice friends*"

What influenced their transitions were the themes of: 1) realization that there was no one else to provide the care, 2) access to information, 3) acknowledging the importance of self-care, 4) love for the person they were caring for and 5) the disease process. They also described what would have helped them: 1) the support of family and friends, 2) access to appropriate services and knowledge, 4) empathic and understanding health care professionals. Participants also felt it was important for researchers to know of their many frustrations and their gratitude for the support they did receive.

### Relationships among demographic variables, hope and quality of life

The mean HHI score was 37.4 (SD 4.94) out of a possible 48 and the WHOQOL BREF overall quality of life score was 3.75 (SD 0.5) out of a possible 5. The results of the multivariate analysis are briefly described below:

#### 1) Overall quality of life (Table 3)

Subjects who had higher hope scores (p < 0.0001) (HHI factor 1: temporality and future-cognitive-temporal dimension of hope) and dealt with their transitions by actively seeking out knowledge and assistance (p = 0.02) had higher overall quality of life scores (question 1 on the WHOQOLBREF).

**Table 3:** Factors associated with overall quality of life

QOL Question 1	Univariate		95% CI		Multivariate		95% CI	
**Variables**	**B**	**SE**	**Lower**	**Upper**	**B**	**SE**	**Lower**	**Upper**
Age	0.00	0.01	-0.02	0.02				
Gender								
	0.17	0.27	-0.36	0.70				
Marital Status								
	0.27	0.33	-0.38	0.92				
Care provided								
	0.26	0.21	-0.15	0.67				
Income								
	-0.14	0.25	-0.63	0.35				
Education								
	0.07	0.21	-0.34	0.48				
Question1								
1	-0.29	0.54	-1.35	0.77				
2	-0.67	0.73	-2.10	0.76				
3	-0.88	0.68	-2.21	0.45				
4	-0.04	0.55	-1.12	1.04				
5	-0.22	0.59	-1.38	0.94				
Question 2								
1	0.44	0.31	-0.17	1.05	0.44	0.25	-0.05	0.93
2	-0.69^1^	0.38	-1.43	0.05	-0.26	0.34	-0.93	0.41
3	0.04	0.30	-0.55	0.63	0.02	0.25	-0.47	0.51
4	0.67^1^	0.33	0.02	1.32	0.65*	0.27	0.12	1.18
5	-0.36	0.35	-1.05	0.33	-0.27	0.29	-0.84	0.30
Question 3								
1	-1.05^1^	0.60	-2.23	0.13				
2	-0.42	0.52	-1.44	0.60				
3	-0.85	0.60	-2.03	0.33				
4	-0.44	0.48	-1.38	0.50				
5	-0.54	0.52	-1.56	0.48				
Question 4								
1	0.32	0.33	-0.33	0.97				
2	-0.62	0.40	-1.40	0.16				
Question 5								
1	0.03	0.25	-0.46	0.52				
Hope Factor1	0.24^1^	0.04	0.16	0.32	0.24*	0.04	0.16	0.32
Hope Factor 2	0.25^1^	0.06	0.13	0.37				
Hope Factor 3	0.20^1^	0.05	0.10	0.30				
HHI Total Score	0.10^1^	0.02	0.06	0.14				

^1 ^Variables significant at p < 0.10 (Univariate analysis)

^2 ^Borderline significance

* Variables significant at p < 0.05

#### 2) Quality of life: physical (Table 4)

Subjects with the highest physical quality of life (WHOQOLBREF Domain one) scores were those who: a) had help with care giving (p = 0.003), b) lower income (p = 0.002), c) had the highest scores for HHI factor 2 (positive readiness and expectancy; affective-behavioural dimension of hope) (p < 0.0001) and d) felt that: i) self-care (p = 0.01), and ii) the disease process (p = 0.006) influenced their transitions the most.

**Table 4:** Factors associated with physical quality of life (domain 1)

Domain 1	Univariate		95% CI		Multivariate		95% CI	
**Variables**	**B**	**SE**	**Lower**	**Upper**	**B**	**SE**	**Lower**	**Upper**
Age	0.03	0.03	-0.03	0.09				
Gender								
	-0.26^1^	0.80	-1.83	1.31				
Marital Status								
	-1.78^1^	0.93	-3.60	0.04				
Care provided								
	0.99^1^	0.57	-0.13	2.11	-1.94*	0.66	-3.23	-0.65
Income								
	-1.23^1^	0.68	-2.56	0.10	-2.10*	0.66	-3.39	-0.81
Education								
	-1.07^1^	0.57	-2.19	0.05				
Question1								
1	0.84	1.53	-2.16	3.84				
2	-1.33	2.07	-5.39	2.73				
3	-0.36	1.93	-4.14	3.42				
4	0.38	1.55	-2.66	3.42				
5	0.95	1.67	-2.32	4.22				
Question 2								
1	-0.24	0.94	-2.08	1.60				
2	-1.44	1.19	-3.77	0.89				
3	0.33	0.93	-1.49	2.15				
4	0.57	1.04	-1.47	2.61				
5	-0.83	1.10	-2.99	1.33				
Question 3								
1	1.37	1.58	-1.73	4.47	2.34	1.66	-0.91	5.59
2	3.54^1^	1.33	0.93	6.15	3.03*	1.23	0.62	5.44
3	1.83	1.58	-1.27	4.93	2.70	1.44	-0.12	5.52
4	3.05^1^	1.22	0.66	5.44	3.19*	1.17	0.90	5.48
5	2.32	1.33	-0.29	4.93	1.76	1.26	-0.71	4.23
Question 4								
1	-0.85	0.85	-2.52	0.82				
2	0.05	1.04	-1.99	2.09				
Question 5								
1	-0.91	0.76	-2.40	0.58				
Hope Factor1	0.54^1^	0.13	0.29	0.79				
Hope Factor 2	0.64^1^	0.18	0.29	0.99	0.89*	0.20	0.50	1.28
Hope Factor 3	0.20	0.18	-0.15	0.55				
HHI Total Score	0.20^1^	0.05	0.10	0.30				

^1 ^Variables significant at p < 0.10 (Univariate analysis)

^2 ^Borderline significance

* Variables significant at p < 0.05

#### 3) Quality of life: psychological (Table 5)

Subjects with the highest scores in psychological quality of life (WHOQOLBREF Domain 2) were older (p = 0.04), and had the highest scores in HHI factor 1 (temporality and future-cognitive-temporary dimension of hope) (p < 0.0001) and HHI factor 3 (interconnectedness, affiliative-contextual dimensions of hope) (p = 0.05).

**Table 5:** Factors associated with psychological quality of life (domain 2)

Domain 2	Univariate		95% CI		Multivariate		95% CI	
**Variables**	**B**	**SE**	**Lower**	**Upper**	**B**	**SE**	**Lower**	**Upper**
Age	0.06^1^	0.02	0.02	0.10	0.03^2^	0.02	-0.01	0.07
Gender								
	-1.08	0.69	-2.43	0.27				
Marital Status								
	-1.02	0.83	-2.65	0.61				
Care provided								
	0.34	0.52	-0.68	1.36				
Income								
	-0.25	0.59	-1.41	0.91				
Education								
	0.82	0.51	-0.18	1.82				
Question1								
1	0.71	1.33	-1.90	3.32				
2	0.67	1.79	-2.84	4.18				
3	0.47	1.67	-2.80	3.74				
4	1.68	1.34	-0.95	4.31				
5	0.21	1.44	-2.61	3.03				
Question 2								
1	0.20	0.81	-1.39	1.79				
2	-1.71^1^	1.02	-3.71	0.29				
3	0.61	0.80	-0.96	2.18				
4	0.58	0.89	-1.16	2.32				
5	-0.93	0.94	-2.77	0.91				
Question 3								
1	-2.80^1^	1.40	-5.54	-0.06				
2	-0.53	1.18	-2.84	1.78				
3	-1.47	1.40	-4.21	1.27				
4	-0.32	1.08	-2.44	1.80				
5	-0.53	1.18	-2.84	1.78				
Question 4								
1	-1.19	0.78	-2.72	0.34				
2	-0.67	0.95	-2.53	1.19				
Question 5								
1	0.69	0.63	-0.54	1.92				
Hope Factor1	0.80^1^	0.09	0.62	0.98	0.67*	0.11	0.45	0.89
Hope Factor 2	0.82^1^	0.13	0.57	1.07				
Hope Factor 3	0.61^1^	0.12	0.37	0.85	0.22^2^	0.12	-0.02	0.46
HHI Total Score	0.33^1^	0.04	0.25	0.41				

^1 ^Variables significant at p < 0.10 (Univariate analysis)

^2 ^Borderline significance

* Variables significant at p < 0.05

#### 4) Quality of life: social relations (Table 6)

Subjects with the highest quality of life in social relations scores (WHOQOLBREF Domain 3) were those with the highest scores in: HHI factor 3 (p < 0.0001) (interconnectedness, affinitive-contextual dimensions of hope).

**Table 6:** Factors associated with relational quality of life (domain 3)

Domain 3	Univariate		95% CI		Multivariate		95% CI	
**Variables**	**B**	**SE**	**Lower**	**Upper**	**B**	**SE**	**Lower**	**Upper**
Age	0.07	0.03	0.01	0.13				
Gender								
	0.54	1.04	-1.50	2.58				
Marital Status								
	-1.22	1.23	-3.63	1.19				
Care provided								
	-1.34^1^	0.73	-2.77	0.09				
Income								
	-1.27	0.85	-2.94	0.40				
Education								
	-0.55	0.76	-2.04	0.94				
Question1								
1	1.87	1.94	-1.93	5.67				
2	0.44	2.62	-4.70	5.58				
3	1.33	2.45	-3.47	6.13				
4	2.50	1.97	-1.36	6.36				
5	-0.27	2.11	-4.41	3.87				
Question 2								
1	1.00	1.19	-1.33	3.33				
2	-1.70	1.50	-4.64	1.24				
3	0.91	1.18	-1.40	3.22				
4	2.08	1.31	-0.49	4.65				
5	-0.39	1.39	-3.11	2.33				
Question 3								
1	-4.80^1^	1.89	-8.50	-1.10				
2	0.28	1.59	-2.84	3.40				
3	-3.47^1^	1.89	-7.17	0.23				
4	-0.96	1.47	-3.84	1.92				
5	-0.50	1.59	-3.62	2.62				
Question 4								
1	-2.50^1^	1.11	-4.68	-0.32				
2	-0.10	1.37	-2.79	2.59				
Question 5								
1	0.14	0.93	-1.68	1.96				
Hope Factor1	0.79^1^	0.17	0.46	1.12				
Hope Factor 2	0.91^1^	0.22	0.48	1.34				
Hope Factor 3	0.81^1^	0.19	0.44	1.18	0.81*	0.19	0.44	1.18
HHI Total Score	0.33^1^	0.07	0.19	0.47				

^1 ^Variables significant at p < 0.10 (Univariate analysis)

^2 ^Borderline significance

* Variables significant at p < 0.05

#### 5) Quality of life: environment (Table 7)

Subjects with the highest environmental quality of life scores (WHOQOLBREF Domain 4) were those who had the highest HHI factor 1 scores (p < 0.0001).

**Table 7:** Factors associated with environmental quality of life (domain 4)

Domain 4	Univariate		95% CI		Multivariate		95% CI	
**Variables**	**B**	**SE**	**Lower**	**Upper**	**B**	**SE**	**Lower**	**Upper**
Age	0.06	0.02	0.02	0.10				
Gender								
	-0.57	0.71	-1.96	0.82				
Marital Status								
	-1.48^1^	0.83	-3.11	0.15				
Care provided								
	0.14	0.52	-0.88	1.16				
Income								
	-1.31^1^	0.60	-2.49	-0.13				
Education								
	-0.83	0.52	-1.85	0.19				
Question1								
1	-1.05	1.35	-3.70	1.60				
2	-1.33	1.82	-4.90	2.24				
3	-2.25	1.70	-5.58	1.08				
4	-0.93	1.36	-3.60	1.74				
5	-2.70^1^	1.47	-5.58	0.18				
Question 2								
1	-0.40	0.80	-1.97	1.17				
2	-0.01	1.01	-1.98	1.97				
3	0.72	0.79	-0.83	2.27				
4	1.17	0.88	-0.55	2.89				
5	-0.13	0.93	-1.95	1.69				
Question 3								
1	-3.00^1^	1.36	-5.67	-0.33				
2	0.78	1.14	-1.45	3.01				
3	-1.70	1.36	-4.37	0.97				
4	-0.27	1.05	-2.33	1.79				
5	-0.62	1.14	-2.85	1.61				
Question 4								
1	0.08	0.78	-1.45	1.61				
2	-0.30	0.95	-2.16	1.56				
Question 5								
1	-1.39	0.65	-2.66	-0.12	-1.13^2^	0.60	-2.31	0.05
Hope Factor1	0.63^1^	0.11	0.41	0.85	0.51*	0.14	0.24	0.78
Hope Factor 2	0.65^1^	0.15	0.36	0.94				
Hope Factor 3	0.53^1^	0.13	0.28	0.78				
HHI Total Score	0.25^1^	0.05	0.15	0.35				

^1 ^Variables significant at p < 0.10 (Univariate analysis)

^2 ^Borderline significance

* Variables significant at p < 0.05

In summary HHI scores were a predictor variable for overall quality of life, and each WHOQOL-BREF quality of life domain. Findings for other demographic and transition variables were not statistically significant.

## Discussion

Hope played a significant role in the subjects' perceptions of overall quality of life as well as physical, psychological, social relations and environmental domains of quality of life. This finding supports the results of Irvin and Acton's [19] study of hope, stress and wellbeing in which hope had a positive significant relationship with wellbeing of women who were family caregivers of persons with AD. Our study included both male and female caregivers, as such adds to Irvin and Acton's findings by suggesting that hope is a significant factor for both female and male caregivers.

Different factors of hope were significant predictors of quality of life domains. Factor 1 hope scores, which reflected questions such as believing each day has potential and having goals, were significantly related to overall quality of life, as well as the psychosocial and environmental quality of life domains. Factor 2, which reflects questions such as seeing possibilities and having a sense of direction, was a significant predictor of physical quality of life scores. The interconnectedness dimension of HHI (factor 3) was a significant predictor of psychological and social relations dimensions of quality of life. Other studies of hope and quality of life in different populations have found significant relationships between these two variables [26,27] but did not delineate which hope factors are the most significant predictors. The increased understanding of hope and quality of life provided by this study can be used as a foundation for the development of hope interventions for family caregivers of persons with AD where critical inputs of the intervention would be targeted to specific quality of life domains.

Subjects who actively sought information and assistance as a way to deal with their transitions had a higher overall quality of life. Thus encouraging family caregivers to take action to get the information they need and to seek help, along with provision of information regarding credible resources may be a way to assist them in dealing with transitions. This finding is similar to an emerging theory of transitions of family caregiving of persons with advanced cancer, which suggests seeking information is an important part of dealing with transitions [25]. While there may be other similarities in the experience of transitions of family caregivers of persons with AD and those with advanced cancer more research is needed to determine if this is the case.

The most significant influences on the participants' physical quality of life were external resources, such as help with care giving, and internal resources such as hope. Moreover those who understood that looking after themselves (self-care) and that the disease process influenced their transitions the most had the highest physical quality of life. These findings are similar to those of other studies of family caregivers. For example the importance of self-care as factor in reducing the effect of caregiver stress on general well-being was reported in a study of 46 family caregivers of persons with dementia [28]. The needs of the caree with regard to assistance from their caregiver and behavioural problems, which is often a function of the disease process, have been found to impact the quality of life of family caregivers of persons with dementia [29]. In our study, participants who perceived that the disease process had an influence on their transitions reported higher physical quality of life. This suggested that understanding that the disease process (not the person's attitude or other factors) influences the carees' behaviours and needs, may help caregivers deal with the carees' changed behaviour.

A surprising finding in our study is that income was inversely related to physical quality of life. This has also been found in other studies where higher levels of income and education are associated with higher levels of strain in family caregivers [30,31]. It could be that family caregivers with higher income and education levels may have higher expectation of themselves as caregivers resulting in higher levels of perceived strain and decreasing levels of physical health. Further research is needed to understand the relationship among higher income and education levels and physical quality of life.

Psychological quality of life (domain 2) was the only domain where age was a significant factor. The older the caregiver and higher the hope scores, the higher the psychological quality of life score. These findings are congruent with gerotranscendence theories that suggest as we age that physical aspects of our quality of life are not as important as our psychological and existential ones [32,33]. It is interesting based on these theories that age was also not a factor influencing the social relations quality of life domain. It may be that our small sample size precluded age from being a significant factor.

### Limitations

There are several limitations to the study. The first is the small sample size.

Although statistically significant findings were reported, additional variables might be significantly related with a larger sample size. As well the poor response rate brings into question the generalizability of the data. The majority of the study participants were caring for a person with AD in their homes. As such the findings reflect the experience of this population. It is possible that there are differences in the transition experience and hope and quality of life based on care setting. Future studies should look at potential differences of those caring for a person at home versus those who are caring for a person in a continuing care setting.

Another limitation is that qualitative data on the transition experience was collected using a survey and so in-depth interviews were not conducted. As well, the qualitative data is a reflection of the study participants and may not reflect the experience of those not in the study. However, without a valid and reliable measure that reflects the transition experience, collecting and transformation qualitative data did provide an understanding of the influence of transitions on quality of life.

It is important to note that even if a family member has able to integrate their transitions into their lives, this does not necessarily mean they have coped well during this process. Coping was not included in this study as a factor influencing quality of life or transitions and is a limitation of the study. However, hoping may in itself may be a form of coping strategy and may account for its significant relationship to quality of life. Future research should include measures of coping when examining quality of life in family caregivers of persons with dementia.

The study was cross-sectional in nature to gain preliminary data on hope, transitions and quality of life. Conducting a longitudinal study with a larger sample would be beneficial for the examination of changes in predictor variables over time.

## Conclusions

This study adds to our understanding of quality of life in family caregivers of persons with dementia. Hope was a significant factor in all aspects of quality of life, underscoring the importance of hope for family caregivers. The mixed method design facilitated understanding the possible role of transitions influencing quality of life. For example, the active engagement of families in seeking information and help, as a way to deal with their transitions, suggests that facilitating this engagement may be of benefit for family caregivers. The findings also suggest many directions for future research, such as increasing our understanding of the processes of transitions and the further development of transition theory for this population. Conducting further research on transitions and quality of life is important to facilitate the development of successful programs that help family caregivers of persons with AD deal with ongoing complex transitions.

## Competing interests

The authors declare that they have no competing interests.

## Authors' contributions

WD participated in the design, collection and analysis of the data, and drafted the manuscript. JS participated in the design of the study and coordinated data collection. SP performed the qualitative data analyses. SG participated in the statistical analysis. All authors read and approved the final manuscript.

## Pre-publication history

The pre-publication history for this paper can be accessed here:

http://www.biomedcentral.com/1471-2318/11/88/prepub
